# Cardiac magnetic resonance of acute atrial ablation injury - impact of catheter-myocardium contact force

**DOI:** 10.1186/1532-429X-16-S1-P149

**Published:** 2014-01-16

**Authors:** Steven E Williams, James Harrison, Lars Ø Bloch, Niels P Andersen, Høgni Dam, Christian Sohns, Henry Chubb, Rashed Karim, Kawal Rhode, Won Yong Kim, Henrik Jensen, Tobias Schaeffter, Reza Razavi, Mark O'Neill

**Affiliations:** 1 Division of Imaging Sciences and Biomedical Engineering, King's College London, London, UK; 2 Department of Cardiology, Aarhus University Hospital, Aarhus, Denmark

## Background

Catheter-myocardium contact force (CF) is a major determinant of ablation lesion generation in the atrium, with higher CF associated with greater energy transfer. Previous publications have suggested that acute atrial injury can be visualized with T2-weighted (T2W) cardiac magnetic resonance (CMR). This study therefore sought to compare T2W images of acute ablation lesions created at low and high CF.

## Methods

Under general anesthesia, femoral venous access was obtained in eight male Göttingen minipigs. In two animals, pre-ablation T2W CMR imaging was performed. Using fluoroscopy, a decapolar reference catheter was placed in the coronary sinus and a force-sensing ablation catheter (SmartTouch, D curve, Biosense Webster) was advanced into the right atrium. Using an electroanatomical mapping system (Carto3-MEM) the geometry of the right atrium was obtained with a 20-pole circular mapping catheter (Lasso Nav, D curve, Biosense Webster). A linear ablation lesion was created from the SVC to the IVC (30W, 48°C, 8 ml/min irrigation). Different target contact forces (>20 g (high force) or <10 g (low force)) were used alternately at the cranial and caudal halves of the ablation line. After the ablation procedure, the animals were immediately transferred for T2W CMR imaging of the ablation lesion. Maximal wall thickness in the posterior right atrium was measured for the caudal and cranial portions of the ablation lines.

## Results

**Figure 1:**
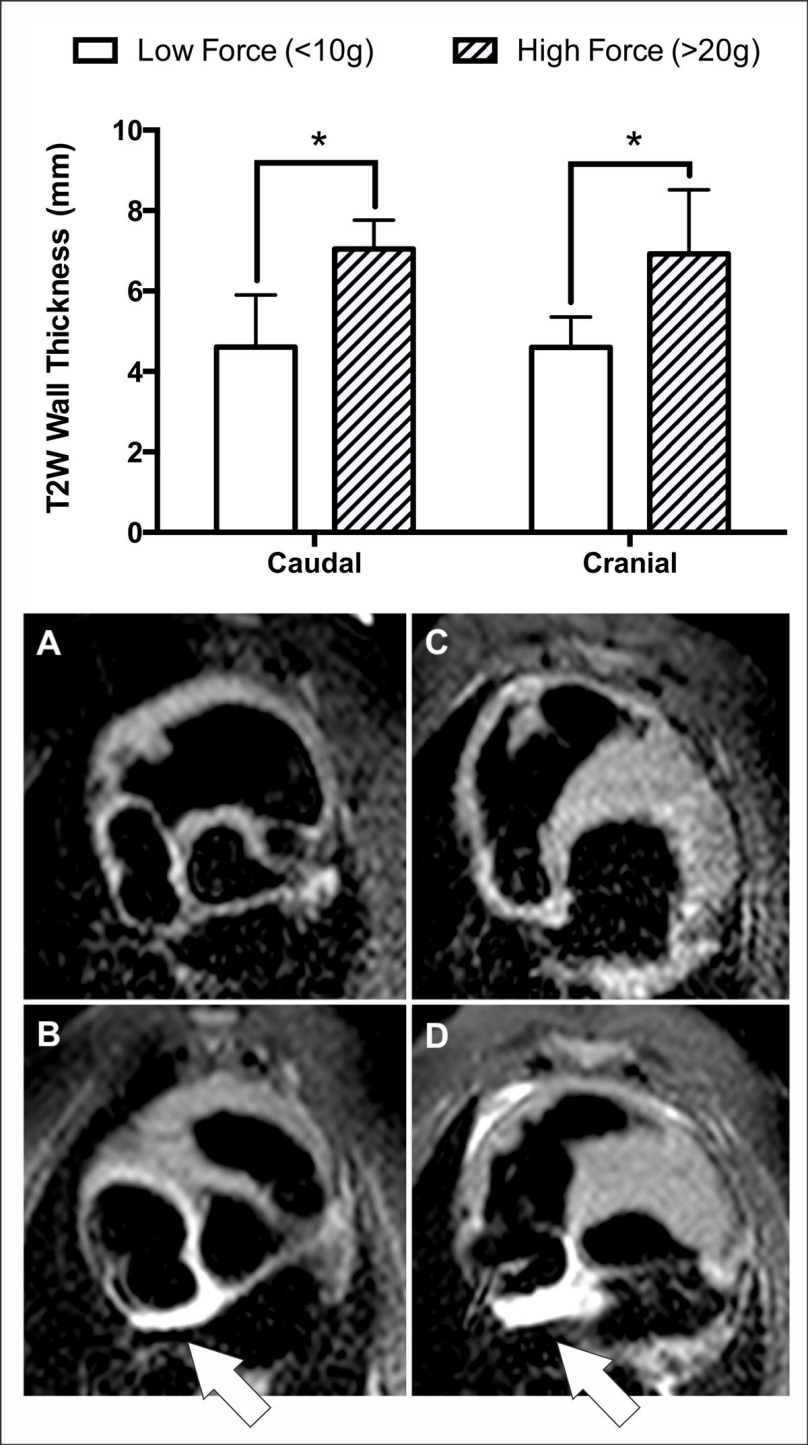
**TOP PANEL Mean T2W wall thickness for high and low ablation forces at the caudal and cranial aspects of the ablation lines**. * Indicates statistical significance. BOTTOM PANEL Typical pre- (A and C) and post-ablation (B and D) T2W images from one animal. Enhancement resulting from ablation is indicated with arrows, for low-force (B) and high force (D) portions of the ablation line.

There was no pre-ablation enhancement seen with T2W imaging in either of the two pre-ablation scans. The mean time from ablation to post-ablation T2W imaging was 1 hour 28 minutes (SD = 19 minutes). Wall thickness measured on post-ablation T2W images was significantly greater in the high CF region than the low CF region, for both the caudal (7.0 mm vs. 4.6 mm; p = 0.016) and cranial (6.9 mm vs. 4.6 mm; p = 0.038) aspects of the ablation line (see Figure 1).

## Conclusions

Wall thickness measured on post-ablation T2W images is significantly greater in areas of ablation performed with high contact force compared to areas of ablation performed with low contact force. These findings indicate that T2W CMR could facilitate the acute assessment of ablation lesion transmurality in patients undergoing atrial ablation procedures.

## Funding

Funding for this research was provided by Biosense Webster.

